# Supplementary material for: An Asymptomatic Patient with Fatal Infertility Carried a Swedish Strain of Chlamydia trachomatis with Additional Deletion in The Plasmid orf1 that Belonged to A Different MLST Sequence Type
Source: Microorganisms. 2019 Jun 28;7(7):187. doi: 10.3390/microorganisms7070187 (PMC6681046; doi:10.3390/microorganisms7070187)
Supplement: Supplementary file 1 [file microorganisms-07-00187-s001.pdf]

Supplemental Information

Table 1. The main sperm characteristics of the case-patient, Saratov city, 2012.

Sperm indicator	Case-patient	WHO reference limit [1]
Concentration of spermatozoa progressive	20%	>32%
non-progressive	27%	
immotile	53%	>50%
vitality	35%	>58%
Total volume	3 ml	≥ 1,5 ml
pH	7.8	≥7,2
Sperm concentration, spermatozoa per ml	12×10^6	15×10^6
